# Back to the future, again: greater leadership, collaboration and accountability to accelerate progress to end TB

**DOI:** 10.1186/s12916-018-1165-9

**Published:** 2018-09-19

**Authors:** Diana Weil, Kristijan Marinkovic, Tereza Kasaeva

**Affiliations:** World Health Organization Global TB Programme, Geneva, Switzerland

## Abstract

A first UN General Assembly high-level meeting on the fight against tuberculosis (TB) will take place on September 26, 2018. It offers the opportunity to forge new concrete commitments and action needed to finance and deliver universal access to care and prevention, to address equity and social determinants of TB, and to pursue robust research and innovation. These are all needed to accelerate declines in TB mortality, incidence, and social and economic burden of the disease. This Commentary looks at leadership precedents in action against TB and highlights opportunities for bolder accountability and collaboration, especially at the country level, to stimulate action and impact.

## Background

Tuberculosis (TB) is caused by the bacillus *Mycobacterium tuberculosis*. It typically affects the lungs and is almost exclusively an airborne infection, with one-quarter of the world’s people being infected. It is the tenth leading cause of death worldwide and the leading cause from a single infectious agent. The World Health Organization (WHO) estimates that TB caused 1.6 million deaths in 2017, including 300,000 among people living with HIV; further, there were 10 million cases of TB that year, with 558,000 involving resistance to rifampicin, the most effective first-line drug, including multi-drug resistant TB (MDR-TB) [1]. “MDR-TB is among the greatest antimicrobial resistant threats, and is considered a global public health crisis.” TB has a global reach, with all regions and countries being affected, and two-thirds of new cases presenting in India, China, Indonesia, Philippines, Pakistan, Nigeria, Bangladesh, and South Africa [1].

Political leadership, collaboration, and accountability are among the key requirements to end the TB epidemic by 2030, a target of the United Nations (UN) Sustainable Development Goals (SDGs) [2]. The first-ever UN General Assembly high-level meeting on the fight against tuberculosis – United to End Tuberculosis: An Urgent Global Response to a Global Epidemic – will take place on September 26, 2018 [3]. Leaders appear poised to seize the opportunity to address, with concrete and measurable actions, this top infectious disease killer. Ministerial leadership and new forms of collaboration have been essential in previous gains, but highest-level national leadership and collective accountability will be vital given the ambition of ending TB in less than 15 years.

### Precedents in political action

The burden of TB in what are today’s high-income countries led leaders to establish regional and global public health agencies in the early half of the last century. TB immunization efforts were among the first mass public health campaigns to reach millions of people. From the 1950s, large-scale access to TB drug treatment screening and prevention programs, where it occurred, was supported by specified disease control policies, regulations, supply and delivery models. Community mobilization for poverty alleviation and social justice led to some advances in legal and social action against pernicious determinants of TB. In many settings, tuberculosis service financing was driven by hard-won central and regional/state public health budgets bolstered by non-governmental campaigns [4]. In Japan, the foundation of the national universal health financing scheme was framed in part to respond to the large burden of tuberculosis [5].

From the late 1970s, weakened financing for public health systems, the emergence of HIV/AIDS and MDR-TB, and increasing migration led to the resurgence of TB in some higher-income countries, with renewed recognition of the scope and scale of epidemics in low- and middle-income countries and the need to respond through global coordinated effort [6]. WHO announced that TB was a global emergency in 1993. Major actions over subsequent decades included new WHO TB control strategies, targets, guidance, and measurement, as well as special attention to TB/HIV and MDR-TB together with stronger domestic, bilateral, and multilateral financing [7]. Global collaboration improved, with the new UN Millennium Development Goals, the Stop TB Partnership/Global Drug Facility, the Global Fund to Fight AIDS, TB and Malaria, research partnerships, and some ministerial meetings. Before 2015, the global epidemic of TB had turned around, and 54 million lives were saved from 2000 to 2017 [1] and TB investment was seen among development researchers and officials as a ‘best buy’ [8].

Yet, the ongoing TB burden is unacceptable for a preventable and curable disease. Based on population-based and health facility surveys, WHO estimates that 3.6 million new TB cases in 2017 went unreported to public health systems and either missed out on care or received care of unknown quality in public or private sectors [1]. Vulnerable and marginalized groups are most likely to be missed. Additionally, treatment programs carry direct and indirect costs and are often not people friendly. Likewise, health system weaknesses are profound in many regions, and efforts to engage civil society have been far from adequate. TB mortality and incidence reductions are slow; the MDR-TB crisis persists, with only one in four people receiving the required treatment, a 55% treatment success rate, and slow access to new diagnostics, and drugs [1].

### New political momentum

The WHO End TB Strategy was adopted to guide accelerated action from 2016, aligned with the SDGs. It set three targets for 2030, namely 90% reduction in deaths, 80% reduction in incidence, and zero catastrophic costs for patients and their families, along with the associated strategic actions. The Stop TB Partnership Global Plan to End TB 2016–2020 proposed the financing framework needed to implement the Strategy and reach targets, as well as the paradigm shift in mind-set needed to make it happen [9]. However, WHO reports that investments and actions thus far fall short of those needed, with a gap of US$ 3.5 billion in 2018 in investment in TB interventions in low- and middle-income countries. The Treatment Action Group reports a US$ 1.3 billion annual gap in TB research financing [10]. Yet, until recently, TB has not been addressed by government leaders, unlike some other global health concerns.

Action at the highest political level is emerging, with recognition of the profound threat of antimicrobial resistance, of the fact that TB has surpassed HIV/AIDS as the greatest infectious killer, and that research is needed for new tools to end TB. Related messages have been sent forth, for example, in the statements of the G20 [11], the Asia-Pacific Economic Cooperation [12], BRICS (Brazil, Russia, India, China, South Africa) [13], and European Parliament leaders [14]. WHO’s new leadership has advanced attention at higher political levels on global health, and engaged with civil society and other stakeholders to assert its commitments to drive greater collaboration to end TB. The WHO Global Ministerial Conference, Ending Tuberculosis in the Sustainable Development Era: A Multisectoral Response, held in November 2017 [15], and its Moscow Declaration to End TB [2], endorsed by ministers and other officials of nearly 120 countries, framed priorities for urgent action and to inform the upcoming UN high-level meeting. The actions included driving the TB response within the SDG Agenda with universal access to care and prevention, sufficient and sustainable financing, intensified research and innovation, and multisectoral accountability. Additionally, the Delhi End TB Summit of Southeast Asian countries in March 2018 [16] and July’s African Union Assembly [17] set new specific commitments.

In order to help enable the universal access to TB treatment needed to end TB, WHO, the Stop TB Partnership, and the Global Fund to Fight AIDS, TB and Malaria have launched an initiative, known as ‘FIND. TREAT. ALL #ENDTB’, with the aim of mobilizing and supporting countries to enable the treatment of 40 million people with TB from 2018 to 2022 [18]. Additionally, the Stop TB Partnership has put forward five ‘Key Asks’ [19] to the leaders participating in the UN high-level meeting largely aligned with the actions called for in the Moscow Declaration [2]. The ‘Key Asks’ include an appeal for US$ 13 billion in TB care and prevention investment annually up to 2022 and US$ 2 billion in annual research investment. The Global TB Caucus of parliamentarians put forward a related position statement and advocacy effort [20].

### Strengthened accountability and action at country level

Driving multisectoral accountability is among the topics set to be addressed at the high-level meeting. In the Moscow Declaration [2], ministers called for WHO to develop, in consultation with Member States and partners, a multisectoral accountability framework. WHO pursued background review of related experiences in global health and other fields and held consultations. The World Health Assembly in May 2018 called for the further development of the framework, as well as its adaptation and use at country level. The draft framework proposes a cycle of efforts at national, regional, and global levels, namely commitments, actions, monitoring and reporting, and review. Among the areas viewed as needing most attention is how best to achieve high-level review that drives multisectoral action, involves civil society, and holds all stakeholders accountable [21].

## Conclusion

The General Assembly calls for the UN high-level meeting on TB to arrive at a concise action-oriented political declaration. In that declaration, Member States have an unprecedented opportunity to shift gear. In September 2019, leaders will meet again to address universal health coverage, another essential SDG health target. The success of that meeting will, in part, depend on actions taken subsequently to this upcoming declaration on TB, on a related declaration of a 2018 high-level meeting on non-communicable diseases, and on the 2016 meetings on HIV/AIDS [22] and antimicrobial resistance [23].

In 1993, with the resurgence of TB at that time, the London School of Hygiene and Tropical Medicine hosted a forum under an apt title, “Tuberculosis: Back to the Future” [24]. Twenty-five years later, the global leaders meeting at UN headquarters should reflect back and move forward, together, with unprecedented speed.

## Abbreviations

MDR-TB: Multidrug-resistant tuberculosis
SDGs: Sustainable Development Goals
TB: Tuberculosis
UN: United Nations
WHO: World Health Organization

## References

[1] World Health Organization. Global TB Report 2018. Geneva: WHO; 2018.

[2] Moscow Declaration to End TB. Geneva: WHO; 2017. http://www.who.int/tb/Moscow_Declaration_MinisterialConference_TB/en/. Accessed 10 Aug 2018.

[3] United Nations General Assembly. Resolution adopted by the General Assembly on 4 April 2018. A/RES/72/268. Scope, Modalities, Format and Organization of the High Level Meeting on the Fight Against Tuberculosis. http://www.un.org/en/ga/search/view_doc.asp?symbol=A/RES/72/268. Accessed 15 Aug 2018.

[4] Binkin NJ, Vernon AA, Simone PM, McCray E et al. Tuberculosis prevention and control activities in the United States: an overview of the organization of tuberculosis services. Int. J Tuberc Lung Dis. 1999;3(8):663–74(12). 10460098

[5] Takemi Keizo (2016). Proposal for a T-Shaped Approach to Health System Strengthening. Health Systems & Reform.

[6] Raviglione M, Pio A (2002). Evolution of Tuberculosis Control Policies 1948-2001. Lancet.

[7] Uplekar M, Weil D, Lönnroth K, Jaramillo E, Lienhardt C, Dias HM (2015). WHO’s new End TB Strategy. Lancet.

[8] United Nations. 2013. A New Global Partnership: Eradicate Poverty and Transform Economies Through Sustainable Development. https://www.un.org/sg/sites/www.un.org.sg/files/files/HLP_P2015_Report.pdf. Accessed 10 Sept 2018.

[9] Stop TB Partnership, UNOPS. The Paradigm Shift 2016–2020: Global Plan to End TB. Geneva: Stop TB Partnership, UNOPS, 2015. http://www.stoptb.org/assets/documents/global/plan/GlobalPlanToEndTB_TheParadigmShift_2016-2020_StopTBPartnership.pdf (Accessed 4 Sept 2018)

[10] Treatment Action Group 2017. The Ascent Begins: Tuberculosis Research Funding Trends, 2005–2016. http://treatmentactiongroup.org/sites/default/files/TB_FUNDING_2017_final.pdf. Accessed 16 Aug 2018.

[11] The G20 Leaders’ Declaration “Shaping an Interconnected World”, adopted in Hamburg on 7–8 July 2017. https://www.g20germany.de/Content/EN/_Anlagen/G20/G20-leaders-declaration.pdf?__blob=publicationFile&v=11. Accessed 13 Aug 2018.

[12] APEC. Joint Statement of the 7th APEC High-Level Meeting on Health & the Economy, 23–24 August 2017, Viet Nam. https://www.apec.org/Meeting-Papers/Sectoral-Ministerial-Meetings/Health/2017_health_him. Accessed 10 Aug 2018.

[13] BRICS. The BRICS Leaders Xiamen Declaration, Adopted on 4 September 2017, in Xiamen, decided to set up “The Tuberculosis Research Network”. https://www.brics2017.org/English/Documents/Summit/201709/t20170908_2021.html. Accessed 10 Aug 2018.

[14] European Parliament. Resolution on the EU’s Response to HIV/AIDS, Tuberculosis and Hepatitis C, 2017/2576 (RSP), on 5 July 2017. http://www.europarl.europa.eu/oeil/popups/ficheprocedure.do?lang=en&reference=2017/2576(RSP)#tab-0. Accessed 10 Aug 2018.

[15] First WHO Global Ministerial Conference, Ending Tuberculosis in the Sustainable Development Era: A Multisectoral Response, http://www.who.int/conferences/tb-global-ministerial-conference/en/ Accessed 4 Sept 2018.

[16] Statement of Action made by Member States of the WHO South-East Asia Region at the Delhi End-TB Summit. http://www.searo.who.int/tb/statement-action-who-sear-end-tb-summit.pdf?ua=1. Accessed 10 Sept 2018.

[17] African Union. Assembly of the Union. Thirty-First Ordinary Session. 1–2 July 2018, Nouakchott, Mauritania. https://au.int/sites/default/files/decisions/34634-assembly_au_dec_690_-_712_xxxi_e.pdf. Accessed 4 Sept 2018

[18] Seventy-first World Health Assembly Commits to Accelerate Action to End TB. http://www.who.int/tb/features_archive/71-world-health-assembly-commits-end-TB/en/. Accessed 13 Aug 2018.

[19] Stop TB Partnership. Key Asks from TB Stakeholders & Communities. 2018. http://www.stoptb.org/global/advocacy/unhlm_asks.asp#Asks.

[20] Global TB Caucus. Global TB Caucus position paper on the High-Level Meeting of the General Assembly on tuberculosis. 2018. https://docs.wixstatic.com/ugd/309c93_1ecb3cff35c544beb16d34592022c351.pdf. Accessed 4 Sept 2018.

[21] A Draft Multisectoral Accountability Framework to Accelerate Progress to End TB. 2018 World Health Assembly. http://apps.who.int/gb/ebwha/pdf_files/WHA71/A71_16Add1-en.pdf. Accessed 4 Sept 2018.

[22] United Nations General Assembly. HIV Political Declaration on HIV and AIDS: On the Fast Track to Accelerating the Fight against HIV and to Ending the AIDS Epidemic by 2030. A/RES/70/266, adopted on 8 June 2016. http://www.un.org/en/ga/search/view_doc.asp?symbol=A/RES/70/266. Accessed 13 Aug 2018.

[23] United Nations General Assembly. Political Declaration of the High-level Meeting of the General Assembly on Antimicrobial Resistance. A/RES/71/3, adopted on 5 October 2016. http://www.un.org/en/ga/search/view_doc.asp?symbol=A/RES/71/3. Accessed 12 Aug 2018.

[24] Porter JDH, McAdam KPWJ (1994). Tuberculosis: Back to the Future. London School of Hygiene & Tropical Medicine Third Annual Public Health Forum.

